# A recombinant adenovirus vector containing the *synNotch* receptor gene for the treatment of triple-negative breast cancer

**DOI:** 10.3389/fonc.2023.1147668

**Published:** 2023-03-29

**Authors:** Ruhan A, Naoto Kunimura, Shoko Tominaga, Erika Hirata, Shunya Nishioka, Misato Uesugi, Rion Yamazaki, Hideto Ueki, Koichi Kitagawa, Masato Fujisawa, Toshiro Shirakawa

**Affiliations:** ^1^ Department of Advanced Medical Science, Kobe University Graduate School of Science, Technology and Innovation, Kobe, Japan; ^2^ Division of Urology, Kobe University Graduate School of Medicine, Kobe, Japan

**Keywords:** adenovirus, breast cancer, gene therapy, cancer stem cells, HIF

## Abstract

Triple-negative breast cancer (TNBC) is known as the most difficult molecular subtype of breast cancer to treat. Recent studies revealed that cancer stem cells (CSCs) play a critical role in TNBC recurrence and metastasis. In this study, we developed a recombinant replication-deficient adenoviral vector (Ad-CD44-N-HIF-3α4), which contains a gene encoding a synthetic Notch (synNotch) receptor composed of the extracellular domain of CD44 (CD44-ECD) and the hypoxia-inducible factor (HIF)-3α4 connected by the Notch core regulatory region. CD44 is a transmembrane glycoprotein and known as a CSC marker in breast cancer and other malignancies. HIF-3α4 is a dominant-negative regulator of HIF-1α and HIF-2α and inhibits hypoxia-inducing effect. Both CD44 and HIF signals contribute cancer stemness and maintaining CSCs in breast cancer. The CD44-ECD in the synNotch receptor acts as the CD44 decoy receptor, and after a ligand such as a hyaluronic acid binds to the CD44-ECD, HIF-3α4 is released from the Notch core domain. We performed an *in vivo* study using a mouse xenograft model of MDA-MB-231, a highly invasive TNBC cell, and confirmed the significant antitumor activity of the intratumoral injections of Ad-CD44-N-HIF3α4. Our findings in this study warrant the further development of Ad-CD44-N-HIF3α4 for the treatment of patients with TNBC.

## 1 Introduction

Breast cancer can be categorized into molecular subtypes by genetic information including the status of estrogen receptor (ER) and progesterone receptor (PR) and human epidermal growth factor receptor 2 (HER2) (1). In most studies the molecular subtypes of breast cancer are divided into four major groups: luminal A (ER-positive, PR-positive and HER2-negative), luminal B (ER-positive, PR-negative and HER2 positive), HER2 positive (ER-negative, PR-negative and HER2 positive), and triple negative breast cancer (TNBC, ER-negative, PR-negative and HER2 negative) (2). Hormone therapy is considered effective against the luminal A and luminal B molecular subtypes, and molecular (HER2) targeted therapy is considered effective against the luminal B and HER2 positive molecular subtypes (3). However, TNBC is refractory to both hormone therapy and HER2 targeted therapy because of the deficiency of target receptors (4, 5). Therefore, TNBC is recognized as the most difficult molecular subtype of breast cancer to treat. Establishing an effective therapeutic modality for TNBC is an urgent unmet need.

**Figure 1:**
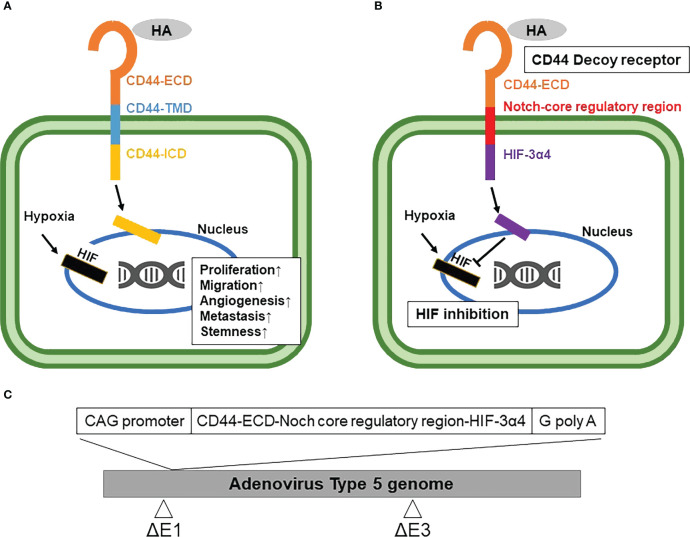
Mechanism of synthetic Notch (synNotch) receptor (CD44-N-HIF3α4) and construction of Ad-CD44-N-HIF3α4 **(A)** CD44 protein: Hyaluronic acid (HA), a ligand of CD44-extracellular domain (CD44-ECD), initiates the CD44 signaling cascade and then induces the cleavage of the intracellular domain of CD44 (CD44-ICD). CD44-ICD moves into the cell nucleus to promote tumor cell proliferation, migration, angiogenesis, and metastasis. **(B)** synNotch receptor (CD44-N-HIF3α4): Hypoxia-inducible factor (HIF) in hypoxic tumor microenvironments also activates the transcription of various hypoxia target genes promoting tumorigenesis and cancer stemness. Ad-CD44-N-HIF3α4 contains a gene encoding a synNotch receptor (CD44-N-HIF3α4) composed of the CD44-ECD and HIF-3α4 connected by the Notch core regulatory region. CD44-ECD in the synNotch receptor acts as a decoy receptor for the endogenous CD44 protein in the tumor microenvironment. The signal of CD44-ligands such as HA is converted *via* the Notch core regulatory region at the cell membrane and inhibits hypoxia-induced responses by HIF-3α4, which is released from synNotch receptor. **(C)** Construction of recombinant replication-deficient adenoviral vector, Ad-CD44-N-HIF3α4 A synthetic fusion gene encoding a synNotch receptor composed of the extracellular domain of CD44 (CD44-ECD) and HIF-3α4 connected by the Notch core regulatory region was introduced into the ΔE1 region of the adenovirus type 5 vector to construct a recombinant replication-deficient adenoviral vector, Ad-CD44-N-HIF3α4. HA, Hyaluronic acid; ECD, Extracellular Domain; TMD, Transmembrane Domain; ICD, Intracellular Domain; HIF, Hypoxia-inducible factor.

Currently, a conventional cytotoxic chemotherapy comprising taxane, anthracycline and/or platinum is the only available option for the systemic treatment of TNBC (6). Although about 20% of TNBC patients achieve a pathological complete response (pCR) to neoadjuvant chemotherapy, most patients suffer early recurrence and metastasis after the initial chemotherapy (7). It is well known that cancer stem cells (CSCs) play an important role in the acquisition of chemoresistance (8, 9), and thus the development of novel TNBC therapies targeting breast cancer stem cells (BCSCs) is attracting great attention (10). CD44 is one of the cell surface adhesion receptors for extracellular matrix proteins, including hyaluronic acid (HA), and is also a known cancer stem cell marker in breast cancer (11). In addition, CD44 is a marker mostly expressed on stem cells, but these cells are very heterogeneous in BC, showing different phenotypes. Cancer stem cells in breast cancer are also identified by CD44+/CD24-/low/EpCAM+ and Aldefuor+ (12). Recent studies have indicated the close association of CD44 with the metastatic ability and stemness of breast cancer cells (13). CD44 ligand-receptor signaling activates kinases that are involved in cell proliferation and migration such as proto-oncogene tyrosine-kinase Src (Src), focal adhesion kinase (FAK) and mitogen-activated protein kinase (MAPK) (14, 15). After the ligand-receptor signaling, the intracellular domain of CD44 (CD44-ICD) is cleaved by protease and then transfers into the cell nucleus, while the CD44-ECD is released as free soluble CD44 (16, 17). CD44-ICD, after entering the cell nucleus, promotes tumor cell proliferation, migration, angiogenesis, and metastasis (**Figure 1A**).

Reportedly, cytotoxic chemotherapy agents including taxane, anthracycline and platinum induce the expression of hypoxia-inducible factor-1α (HIF-1α) and increase the subpopulation of BCSCs (18). Indeed, the CD44+/CD24-/low CSC phenotype is associated with the expression oh HIF-1α and poor survival of patients with breast cancer (19). HIF is a heterodimeric complex composed of an oxygen (O_2_)-labile α subunit and a stable β subunit, which is the aryl hydrocarbon receptor nuclear translocator (ARNT). The HIF α subunit includes HIF-1α, HIF-2α and HIF-3α, and the HIF β subunit includes HIF-1β. HIF-1α and HIF-2α form a heterodimer with HIF-1β and then bind to the hypoxia-responsive elements (HREs), which activate the transcription of various hypoxia target genes (20, 21) promoting tumorigenesis and CSC maintenance (**Figure 1A**) (22). HIF-3α exists as multiple splice variants, and some variants including HIF-3α4 inhibit the gene transcriptions mediated by HIF-1α and HIF-2α in a dominant negative fashion (23).

Recent studies revealed that BCSCs play a critical role in TNBC recurrence and metastasis (24, 25). Additionally, an exposure to cytotoxic chemotherapies increases the subpopulation of BSCSs (5). Thus, a novel therapy targeting BCSCs has a great potential to suppress the acquisition of TNBC chemoresistance, recurrence and metastasis (10). It is well known that both CD44 and HIF contribute cancer stemness in the tumor microenvironment (26, 27). To target both CD44 and HIF simultaneously, we constructed a recombinant replication-deficient adenovirus vector (Ad-CD44-N-HIF3α4) containing a gene encoding a synthetic Notch (synNotch) receptor (CD44-N-HIF3α4) composed of the extracellular domain of CD44 (CD44-ECD) and HIF-3α4 connected by the Notch core regulatory region (**Figure 1B**). Ad-CD44-N-HIF3α4 can induce overexpression of the synNotch receptor of CD44-N-HIF3α4 in cancer cells. The CD44-ECD in the synNotch receptor acts as a CD44 decoy receptor in the tumor microenvironment (TME), and after a ligand binds to the CD44-ECD, HIF-3α4 is released from the Notch core regulatory region at the cell membrane to inhibit the hypoxia-induced responses (**Figure 1B**). Notch signaling is highly conserved in multicellular organisms and features signaling through direct interactions among adjacent cells (27). Notch receptors bind Jagged and Delta-like ligands on adjacent cell surfaces, and the ICD of Notch is cleaved by a disintegrin and metalloproteinases (ADAMs) and multiprotein γ-secretase complexes, and then transfers to the nucleus to promote transcription of target genes (28, 29).

In the present study we explored the feasibility of using Ad-CD44-N-HIF3α4 to treat TNBC by *in vitro* and *in vivo* experiments using MDA-MB-231 a human TNBC cells and compared it’s *in vivo* anti-tumor activities with other adenovirus vectors, Ad-SOCS3 (30) and Ad-p53 (31). These Ad-vectors had been intensively investigated in animal or human studies (30. 31) and were used as the control drugs in this study. As the results, we confirmed that Ad-CD44-N-HIF3α4 showed the strongest anti-tumor activity in MDA-MB-231 xenograft model *in vivo* compared to the other Ad-vectors.

## 2 Materials and methods

### 2.1 Cell lines

MDA-MB-231, a human TNBC cell line, was purchased from The European Collection of Cell Cultures (ECACC, Salisbury, UK) and cultured in Leibovitz’s L-15 Medium (FUJIFILM Wako Pure Chemical Corporation, Osaka, Japan) supplemented with 15% fetal bovine serum (FBS; Sigma Aldrich, St. Louis, MO), 100 U/mL penicillin, and 100 μg/mL streptomycin (Nacalai Tesque, Kyoto, Japan) at 37°C. MCF-7, a human breast cancer cell line (Luminal A, ER+, HER2-), HEK293, a human embryonic kidney cell line, and SV-HUC-1, a human uroepithelium cell line were purchased from American Type Culture Collection (ATCC, Manassas, VA) and cultured in Dulbecco’s modified Eagle’s medium (DMEM, FUJIFILM Wako Pure Chemical Corporation) supplemented with 10% FBS, 100 U/mL penicillin, and 100 μg/mL streptomycin at 37°C in a humidified atmosphere of 5% CO_2_. To generate a hypoxia culture condition, we used Anaero Pack 2%, Anaerobic cultivation sets (Mitsubishi Gas Chemical Company, Inc., Tokyo, Japan).

### 2.2 Construction of Ad-CD44-N-HIF3α4

The construction of the *synNotch* receptor fusion gene, *CD44-N-HIF3α4*, was outsourced to GENEWIZ (South Plainfield, NJ, USA). The *synNotch receptor* gene, which had previously been subjected to restriction enzyme treatment with *SwaI* enzyme (TaKaRa Bio Inc., Kusatsu, Japan), electrophoresis, and purification, was used as an insert DNA. The respective domains of the *synNotch* receptor gene were designed based on published articles (32–34), the transmembrane domain search tool TMHMM (http://www.cbs.dtu.dk/services/TMHMM/), and the signal peptide sequence tool SignalP (http://www.cbs.dtu.dk/services/SignalP/). Subsequently, the sequences thereof were determined. The pAxCAwtit2 cosmid vector, which was included in the Adenovirus Dual Expression Kit (TaKaRa Bio Inc.), was used as vector DNA for constructing Ad-CD44-N-HIF3α4. Ad-CD44-N-HIF-3α was constructed by transfecting this vector DNA (**Figure 1C**) to HEK293 cells. The abovementioned procedures were conducted as per the manual of the Adenovirus Dual Expression Kit.

### 2.3 Ad-SOCS3 and Ad-p53

Ad-SOCS3, a replication-deficient recombinant adenoviral vector expressing human *Suppressor of cytokine signalling 3 (SOCS3)* gene, Ad-p53, a replication-deficient recombinant adenoviral vector expressing human *p53* gene, and Ad-LacZ, a replication-deficient recombinant adenoviral vector expressing *β-galactosidase (LacZ)* gene, were constructed by the cosmid-adenoviral DNA terminal protein complex method (35–37). Ad-SOCS3, Ad-p53 and Ad-LacZ were designed to express *SOCS3* gene, *p53* gene and *LacZ* gene, respectively, under the control of the CAG promoter (a modified chicken β-actin promoter with a cytomegalovirus immediate early enhancer) (38). The viruses were amplified in HEK293 cells and purified using CsCL_2_ step gradient ultracentrifugation followed by CsCl_2_ linear gradient ultracentrifugation. The purified viruses were dialyzed against a solution containing 10 mM Tris-HCl (pH 7.5), 1 mM MgCl_2_, and 10% glycerol and stored at -80°C (39). Viral particle and biological titers were determined using a standard plaque-forming assay (37). This study was approved by the Committee for Safe Handling of Living Modified Organisms of Kobe University and carried out according to the committee guidelines.

### 2.4 Flow cytometry

The expressions of coxsackievirus and adenovirus receptor (CAR) and CD44 on the surface of MDA-MB-231 and MCF-7 cells were assessed by flow cytometry. Briefly, the cells (1×10^6^ cells/well) were seeded in 6-well flat bottom culture plates (Corning, Corning, NY) and incubated for 48 hours at 37°C and 5% CO_2_, and then washed with phosphate buffer solution (PBS). Blocking One Histo (Nacalai Tesque, Inc., Kyoto, Japan) was used to conduct 10-minute blocking at room temperature. Cells were re-washed with PBS after blocking. The 100 fold-diluted PE anti-CAR antibody, Clone: RmcB (Catalog#: 05-644, Sigma-Aldrich) or the 100 fold-diluted PE mouse IgG1, κ Isotype Ctrl, Clone: MOPC-21 (Catalog#: 400101, BioLegend, San Diego, CA) for CAR, and the 200 fold-diluted FITC anti-mouse/human CD44 antibody, Clone : IM7 (BioLegend) or the 100 fold-diluted FITC Rat IgG2a, κ Isotype Ctrl, Clone: RTK2758 (Catalog#: 103007, BioLegend) for CD44, was added for a 30-minute reaction on ice. Cells were re-washed with PBS after reaction and 100 fold-diluted BD Pharmingen™ 7-AAD (BD Biosciences, San Diego, CA) was added for a 5-minute reaction on ice under light-resistant conditions. Cells were re-washed with PBS after this reaction. expression of CAR and CD44 were determined by Guava^®^ easyCyte™ (Merck Millipore, Burlington, MA), and data were analyzed with InCyte software.

### 2.5 Real-time reverse transcriptase-polymerase chain reaction

The induction of *HIF-1α* and *vascular endothelial growth factor (VEGF)* mRNAs by hypoxia (2%) in MDA-MB-231 and MCF-7 cells were examined by real-time RT-PCR. Also, gene expressions of *hyaluronan synthase (HAS) 1, HAS2* and *HAS3* mRNAs in MDA-MB-231, MCF-7 and SV-HUC-1 cell lines were examined by real-time RT-PCR. The gene transduction with recombinant adenoviral vectors including Ad-CD44-N-HIF3α4 and the anti-tumor effect obtained thereby were verified by real-time RT-PCR. Briefly, the cells (1×10^6^ cells/well) were seeded in the 6-well flat-bottomed culture plates (Corning Inc.), and cultured overnight at 37°C and 5% CO_2_ under conditions of normoxia (21%) or under conditions of hypoxia (2%). AnaeroPack^®^-Kenki (Mitsubishi Gas Chemical Co., Ltd.) and HA (40-80 kDa; PG Research, Tokyo, Japan) were used to generate the culture condition of hypoxia (2%). The cells were infected with Ad-CD44-N-HIF3α4, Ad-SOCS3, Ad-p53, or Ad-LacZ respectively at a multiplicity of infection (MOI) of 40 pfu/cell. Cells were incubated for another 48 hours and then retrieved to extract total RNA by using NucleoSpin^®^ RNA (TaKaRa Bio). cDNA was synthesized from extracted RNA using the PrimeScript™ RT Regent Kit with gDNA Eraser (TaKaRa Bio Inc.). The primers (**Table 1**), TB Green™ Prime Ex Taq™ (TaKaRa Bio Inc.), and Thermal Cycler Dice^®^ Real Time System (TaKaRa Bio Inc.) were used to conduct real-time RT-PCR prior to analyses according to the ΔΔCt method.

**Table 1:** Primer sequences for real-time RT-PCR.

Genes	Sequences
*HIF-1α*	Forward: 5’-TATGAGCCAGAAGAACTTTAGGC-3’
	Reverse: 5’-CACCTCTTTTGGCAAGCATCCTG-3’
*VEGF*	Forward: 5’-GGGCCTCCGAAACCATGAAC-3’
	Reverse: 5’-CAAGGCTCCAATGCACCCAA-3’
*HAS1*	Forward: 5’-GGAATAACCTCTTGCAGCAGTTTC-3’
	Reverse: 5’-GCCGGTCATCCCCAAAAG-3’
*HAS2*	Forward: 5’-TCGCAACACGTAACGCAAT-3’
	Reverse: 5’-ACTTCTCTTTTTCCACCCCATTT-3’
*HAS3*	Forward: 5’-AACAAGTACGACTCATGGATTTCCT-3’
	Reverse: 5’-GCCCGCTCCACGTTGA-3’
*HIF-3α4*	Forward: 5’-GGGAGACATGGCTTACCTGT-3’
	Reverse: 5’-GCGTACTCTTCATGCGCAAG-3’
*SOCS3*	Forward: 5’-GACCAGCGCCACTTCTTCAC-3’
	Reverse: 5’-CTGGATGCGCAGGTTCTTG-3’
*p53*	Forward: 5’-CAGCCAAGTCTGTGACTTGCACGTAC-3’
	Reverse: 5’-CTATGTCGAAAAGTGTTTCTGTCATC-3’
*Survivin*	Forward: 5’-AGAACTGGCCCTTCTTGGAGG-3’
	Reverse: 5’-CTTTTTATGTTCCTCTATGGGGTC-3’
*CCL2*	Forward: 5’-AAGATCTCAGTGCAGAGGCTCG-3’
	Reverse: 5’-TTGCTTGTCCAGGTGGTCCAT-3’
*Bcl-xL*	Forward: 5’-CCCAGAAAGGATACAGCTGG-3’
	Reverse: 5’-GCGATCCGACTCACCAATAC-3’
*TBP*	Forward: 5’-GCCAGCTTCGGAGAGTTCTGGGATT-3’
	Reverse: 5’-CGGGCACGAAGTCAATGGTCTTTA-3’

### 2.6 Western blotting

MDA-MB-231 and MCF-7 cells were plated in 6-well plates at a density of 5×10^5^ cells/well and incubated at 37°C and 5% CO_2_ for 24 hours. Then cells were infected with Ad-CD44-N-HIF3α4 at 5, 25, 50 and 100 MOIs and incubated an additional 48 hours. These cells were harvested and washed by PBS, then lysed in 8 M urea buffer containing 0.1% dithiothreitol, and protein concentration was determined. Equal amounts of each sample were added into sample buffer (Nacalai Tesque) and heated at 95°C for 5 minutes. The samples were separated by sodium dodecyl sulfate-polyacrylamide gel electrophoresis (SDS-PAGE) and transferred to a polyvinylidene difluoride membrane. After blocking with Blocking One (Nacalai Tesque) 1 hour at room temperature (RT), followed by washing, the membranes were incubated overnight at RT with anti-CD44 (E7K2Y) monoclonal antibody (Catalog#: 37259S, Cell Signaling Technology, Danvers, MA), 1:1000, or anti-beta-actin antibody (Catalog#: sc-47778, Santa Cruz Biotechnology, Dallas, TX), 1:1000. The CD44 antibody was diluted with Can Get Signal Immunoreaction Enhancer Solution (TOYOBO, Osaka, Japan), and beta-actin antibodies was diluted with PBS-Tween 20%. After another washing, membranes were incubated for 1 hour at RT with horse radish peroxidase (HRP) conjugated goat anti-mouse IgG or anti-rabbit IgG 1:1000. Antibody binding to proteins was detected by enhanced chemiluminescence.

### 2.7 Cell proliferation assay

MDA-MB-231 and MCF-7 cells were seeded at a density of 2.0×10³ cells/well in a 96-well plate (Thermo Fisher Scientific, Waltham, MA) and cultured for 24 hours. Then, the cells were treated with 50 MOI of Ad-LacZ or Ad-CD44-N-HIF3α4 and were incubated at 37°C for 5 days at 2% or 21% oxygen concentration. And then, colorimetric reagents Cell Titer 96 Aqueous One Solution Cell Proliferation Assay (Promega, Madison, WI) were added and the absorbance was measured at a wavelength of 490 nm using microplate photometer (Thermo Fisher Scientific). The relative cell proliferation rate was determined by calculating the rate of reduce of the obtained absorbance with cell only as 1.

### 2.8 Transwell migration assay

To investigate the cell migration ability of MDA-MB-231 after transduction of *CD44-Notch-HIF3α4* gene, MDA-MB-231 cells were seeded at a density of 5.0×10^4^ cells/well in a 6-well plate (Corning, Inc.) and cultured for 24 hours. Then, the cells were treated with 50 MOI of Ad-LacZ or Ad-CD44-N-HIF3α4 and were incubated at 37°C for another 24 h at 2% or 21% oxygen concentration. And then those cells were seeded in the insert chamber (1.0×10_4_ cells per chamber) under serum-free condition, and 10% FBS was determined as a chemoattractant in the bottom well of the Transwell^®^ 6.5 mm Polycarbonate Membrane Inserts Pre-Loaded in 24-Well Culture Plates, Pore Size: 8 µm (Corning Inc.) and the cells were incubated for additional 24 h. The migrated cells on the bottom side of membrane were stained with crystal violet and viewed under a microscope. The numbers of migrating cells per field of view were counted using a microscope at ×100 magnification.

### 2.9 Animal studies

An *in vivo* study in mice was conducted to compare the anti-tumor effect of Ad-CD44-N-HIF-3α4 with other adenoviral vectors. Briefly, the mixture of MDA-MB-231 cells (1×10^6^ cells/70 μL) and 70 μL of Matrigel^®^ Matrix Basement Membrane HC (Corning, Inc.) was subcutaneously inoculated into the right lumbar region of 25 female BALB/c-nu/nu mice aged 6 weeks (CLEA Japan, Inc., Tokyo, Japan). Tumor implantation was verified on day 14 after xenografting, and the 25 mice were randomly allocated to 5 treatment groups in blinded manner. The treatment groups were established for a total of 8 intratumoral injections of adenoviral vectors and control on alternate days (days 14, 16, 18, 20, 22, 24, 26, and 28): Ad-CD44-N-HIF3α4, Ad-SOCS3, Ad-p53, and Ad-LacZ (1×10^9^ PFU/50 µL each), and PBS control (50 μL). Tumor diameters were measured 5 times in total, twice weekly, from the start day of injection. The major (L) and minor (W) axes of the tumor were measured to calculate tumor volume according to the formula; (W^2^×L)/2. After treatment completion, tumors were removed and fixed with 4% paraformaldehyde phosphate buffer solution (FUJIFILM Wako Pure Chemical Corporation) for immunohistochemical studies.

### 2.10 Immunohistochemical staining

Tumor tissues were resected and fixed with paraformaldehyde. Paraffin embedded MDA-MB-231 tumor tissue sections were deparaffinized and rehydrated. Antigen retrieval was performed in Bond epitope retrieval buffer (pH6.0; Leica Microsystems, Wetzlar, Germany) at 98°C for 20 minutes. Immunohisochemical staining was performed in an automatic tissue processor (Leica Microsystems Bond) according to the manufacturer’s standard protocol. Briefly, tissue sections were incubated at RT for 15 minutes with anti-CD44 antibody (F10-44-2) (1:100, Catalog#: ab6124, Abcam, Cambridge, UK). After washing, sections were incubated with HRP conjugated secondary antibodies. After washing, sections were incubated with 3,3’-diaminobenzidine (Muto Pure Chemicals Co., Ltd., Tokyo, Japan) and counterstained with hematoxylin. The resulting tissue slides were observed under a BZ-X710 microscope (Keyence, Osaka, Japan).

### 2.11 Statistical analysis

Comparisons between two groups were performed by the student’s t -test and comparisons between multiple groups were performed by one-way ANOVA followed by the Tukey–Kramer method. Differences among experimental groups were considered significant when p<0.05. The sample size of animal study was calculated by the power analysis approach.

### 2.12 Study approval

All experiments and methods were performed in accordance with relevant guidelines and regulations, and all experimental protocols were approved by the committees of the Kobe University Graduate School of Medicine. Specifically, the animal experimental design and procedure were reviewed and approved by the institutional ethics and animal welfare committees of the Kobe University Graduate School of Medicine.

## 3 Results

### 3.1 MDA-MB-231 cells express CAR and CD44 and mRNA expressions of *HIF-1α* and *VEGF* are increased under hypoxia

**Figure 2:**
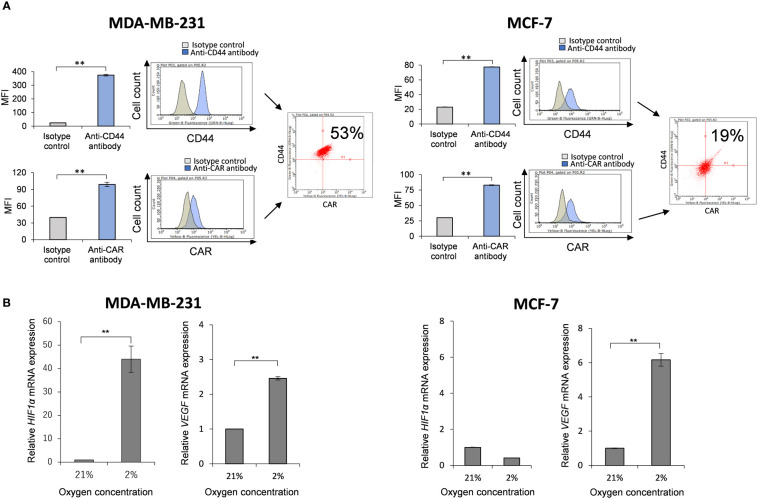
Expressions of CAR and CD44, and induction of *HIF-1α* mRNA by hypoxia in MDA-MB-231 and MCF-7 cells **(A)** The expressions of CAR and CD44 on the cell surface of MDA-MB-231 and MCF-7 cells were determined by flow cytometry. Mean fluorescence intensities (MFI) and their representative histograms are shown. Both CAR and CD44 expressions were significantly higher than their isotype controls (n=3, average ± SE bars, **p< 0.01). However, the CD44 expression in MCF-7 was relatively lower than MDA-MB-231 cells. The upper right corner of the histogram represents CD44 and CAR double positive cells. **(B)** The mRNA expressions of *HIF-1α* and *VEGF* in MDA-MB-231 and MCF-7 cells were measured by real-time RT-PCR under the culture conditions of O_2_ concentrations of 21% and 2%. The mRNA levels of *HIF-1α* and *VEGF* in MDA-MB-231 cells were significantly increased in 2% hypoxia culture condition compared to 21% condition. In addition, mRNA level of *HIF-1α* in MCF-7 cells was not increased in hypoxia, while *VEGF* was significantly increased in hypoxia (n=3, average ± SE bars, **p< 0.01).

Prior to investigation of anti-tumor activity of Ad-CD44-N-HIF-3α4 in MDA-MB-231 (TNBC) cells, we confirmed the expressions of CAR and CD44 on the cell surface and the induction of *HIF-1α* and *VEGF* mRNA by hypoxia. The expression of CAR on the cell surface is strongly correlated with the infectivity of adenovirus type 5 (40), thus confirmation of cell-surface expression of CAR protein is important for the further evaluation of Ad-vectors. The significantly higher expressions of CAR and CD44 compared to each isotype controls were confirmed by flow cytometry (p < 0.01; **Figure 2A**). The CAR and CD44 double positive population of MDA-MB-231 and MCF-7 cells are 53% and 19%, respectively (**Figure 2A**). Also, the mRNA levels of *HIF-1α* and *VEGF* were significantly increased in 2% hypoxia culture condition compared to 21% condition in MDA-MB-231 cells (p < 0.01; **Figure 2B**). These results indicate that MDA-MB-231 cell line is suitable for further investigating the anti-tumor activity of Ad-CD44-N-HIF 3α4. As for the control breast cancer cell line, we employed MCF-7 (Luminal A, ER+, HER2-) cell line in this examination. Although MCF-7 cells expressed CAR and CD44 proteins, the expression of CD44 was relatively lower compared to MDA-MB-231 cells (**Figure 2A**). In addition, mRNA level of *HIF-1α* in MCF-7 cells was not increased in hypoxia, while *VEGF* was significantly increased in hypoxia (**Figure 2B**).

### 3.2 MDA-MB-231 cells express *HAS2* mRNA

**Figure 3:**
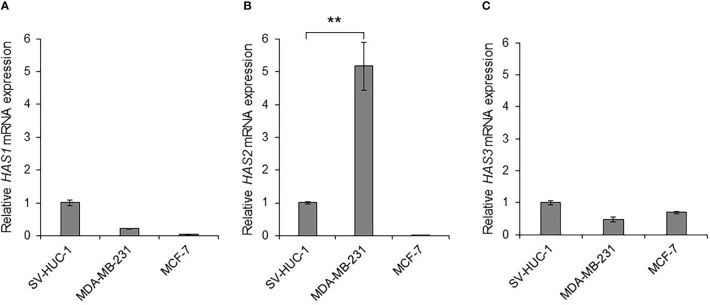
Detection of *hyaluronan synthases (HAS1, HAS2, and HAS3)* mRNA in MDA-MB-231 and MCF-7 cells **(A)** The mRNA expressions of *HAS1*, *HAS2* and *HAS3* in MDA-MB-231 and MCF-7 cells were measured by real-time RT-PCR and compared to those in non-malignant SV-HUC-1 cells. The mRNA expressions of *HAS1* in MDA-MB-231 and MCF-7 cells was not increased compared to those of SV-HUC-1 cells. **(B)** The mRNA expression of *HAS2* in MDA-MB-231 cells was significantly increased compared to that in SV-HUC-1 cells, but not in MCF-7 cells. **(C)** The mRNA expressions of *HAS3* in MDA-MB-231 and MCF-7 cells was not increased compared to that in SV-HUC-1 cells. mRNA levels were standardized by the expression levels of control gene *TATA-binding protein* (*TBP*). (n=3, average ± SE bars, **p< 0.01).

Some types of cancer cells express hyaluronan synthases (HAS1, HAS2, and HAS3), as well as embryonic cells, and HAS2-CD44 signaling is considered to play a vital role in malignant progression (41). We compared the mRNA expressions of *HAS1*, *HAS2* and *HAS3* in MDA-MB-231 and MCF-7 cells to those in non-malignant SV-HUC-1 cells (**Figures 3A–C**). As the result, only *HAS2* mRNA expression in MDA-MB-231 cells was significantly increased compared to SV-HUC-1 cells (p < 0.01; **Figure 3B**), but not in MCF-7 cells.

### 3.3 Ad-CD44-N-HIF3α4 transduces CD44-ECD proteins both in MDA-MB-231 and MCF-7 cells in dose-dependent manner but inhibits the cell growth of only MDA-MB-231 cells

**Figure 4:**
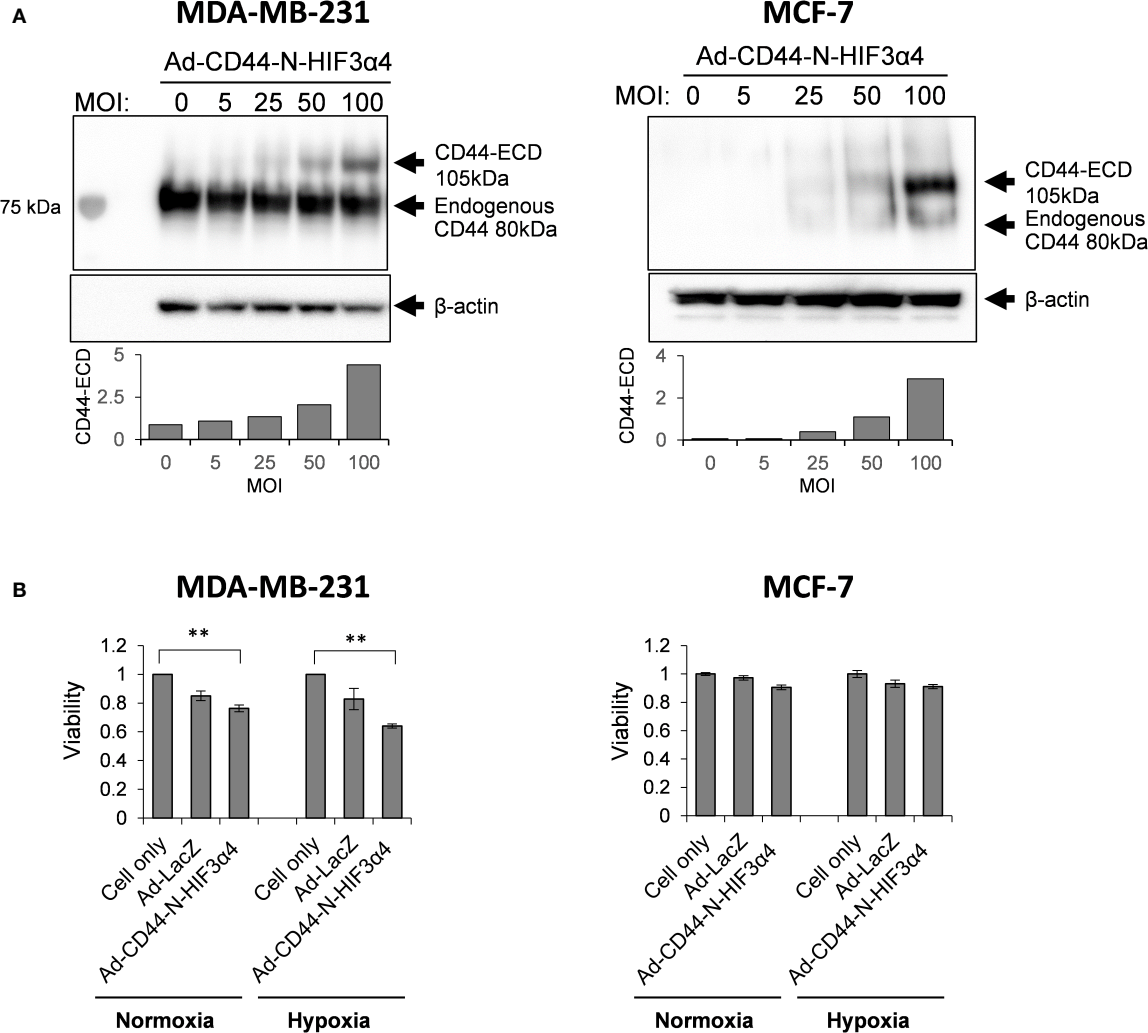
Ad-CD44-N-HIF3α4 transduced CD44-ECD protein in MDA-MB231 and MCF-7 cells in dose-dependent manner but inhibits the cell growth of only MDA-MB-231 cells **(A)** Western blotting using anti-CD44 antibody revealed that MDA-MB-231 highly expressed endogenous CD44 protein (around 80 kDa) but MCF-7 does not express the endogenous CD44 protein. Ad-CD44-N-HIF3α4 could induce the synNotch receptor protein including CD44-ECD in dose dependent manner both in MDA-MB-231 and MCF-7. **(B)** Cell proliferation analysis, Ad-CD44-N-HIF3α4 significantly inhibited the cell growth of MDA-MB-231 cells in normoxia and hypoxia conditions compared to cell only (no treatment) group but did not inhibit the cell growth of MCF-7 cells (n=3, average ± SE bars, **p< 0.01).

To confirm that Ad-CD44-N-HIF3α4 could transduce the CD44-ECD protein, we performed the western botting assay using anti-CD44 antibody in various doses (0, 5, 25, 50, and 100 MOIs) of Ad-CD44-N-HIF3α4. As a result, beside the endogenous CD44 protein, we confirmed the expression of CD44-ECD synthetic fusion proteins in both MDA-MB-231 and MCF-7 cells in dose dependent manners (**Figure 4A**). In addition, the strong expressions of endogenous CD44 proteins were observed in MDA-MB-231 cells, while they were merely seen in MCF-7 cells. Generally, it is well known that MDA-MB-231 (TNBC) cell line has more metastatic characteristics than MCF-7 (Luminal A, ER+, HER2-) cell line (42). Thus, we decided to use the MDA-MB-231 cell line for further evaluations of Ad-CD44-N-HIF3α4 *in vitro* and *in vivo* experiments. Furthermore, in our cell proliferation analysis, Ad-CD44-N-HIF3α4 significantly inhibited the cell growth of MDA-MB-231 cells both in normoxia and hypoxia conditions compared to cell only (no treatment) group but did not inhibit the cell growth of MCF-7 cells (p<0.01; **Figure 4B**).

### 3.4 Ad-CD44-N-HIF3α4 inhibits migration in MDA-MB-231 in hypoxia condition

**Figure 5:**
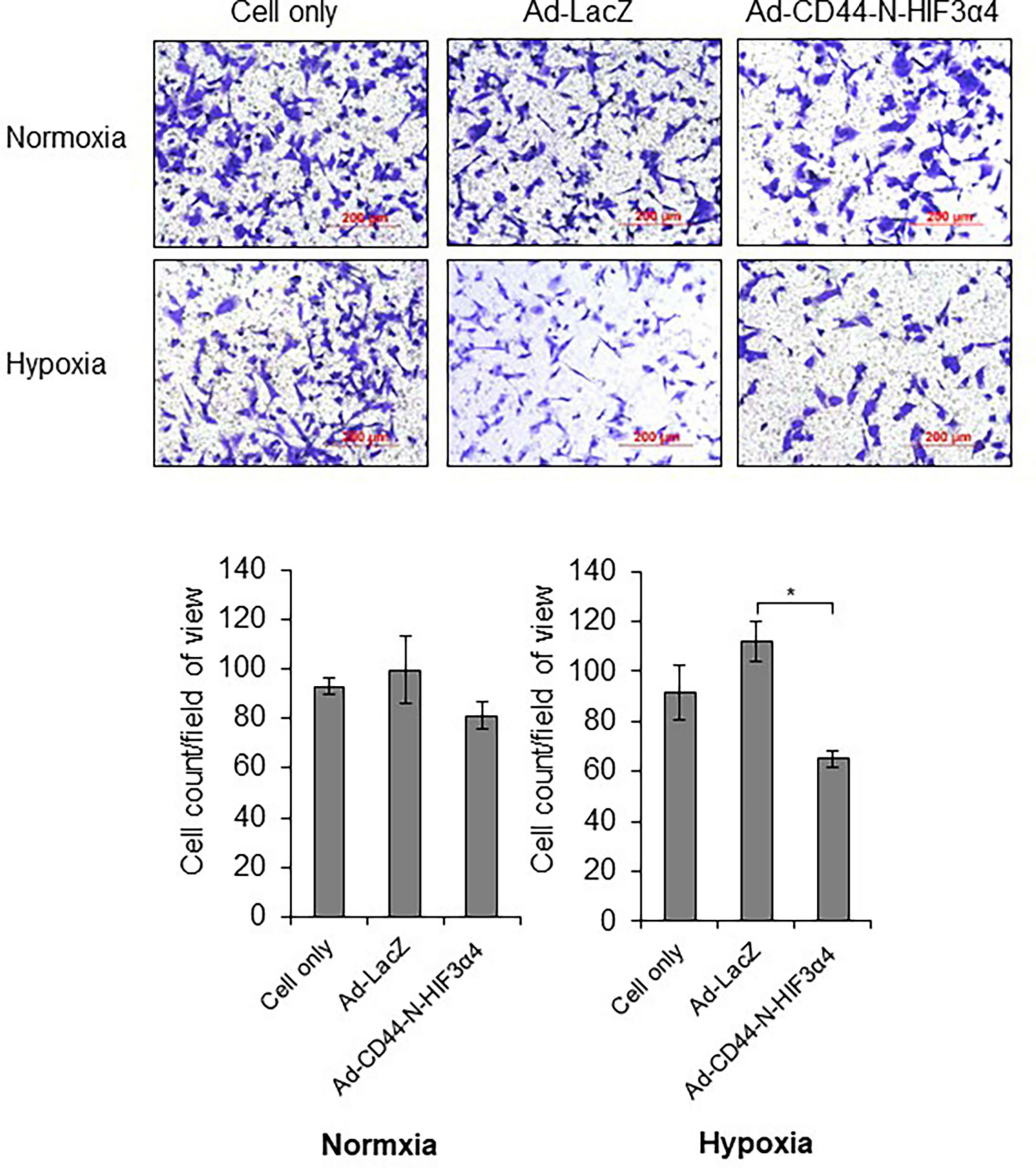
Ad-CD44-N-HIF3α4 inhibited migration in MDA-MB-231 in hypoxia condition, Ad-CD44-N-HIF3α4 significantly inhibited the cell migration of MDA-MB-231 cells in hypoxia condition but not in normoxia condition. Magnification: ×100 (n=3, average ± SE bars, *p< 0.05).

In the transwell migration assay, Ad-CD44-N-HIF3α4 significantly inhibited the cell migration of MDA-MB-231 cells in hypoxia condition but not in normoxia condition (p<0.05; **Figure 5**).

### 3.5 Recombinant adenoviral vectors induced respective gene transductions

**Figure 6:**
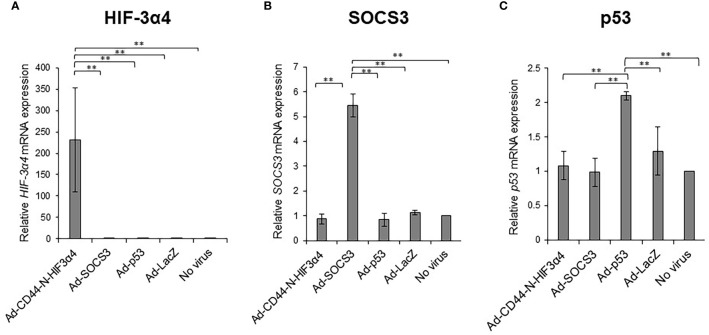
Gene expression of *HIF-3α4*, *SOCS3* and *p53* induced by adenovirus vectors in MDA-MB-231 cells The gene expression of *HIF-3α4* **(A)**, *SOCS3* **(B)** and *p53* **(C)** in MDA-MB-231 cells were measured by real-time RT-PCR after Ad-CD44-N-HIF3α4, Ad-SOCS3, Ad-p53 and Ad-lacZ infections. mRNA levels were standardized by the expression levels of control gene *TATA binding protein* (*TBP*). The significantly increased levels of mRNA expressions of *HIF-3α4* **(A)**, *SOCS3* **(B)** and *p53* **(C)** were observed in cells infected with Ad-CD44-N-HIF3α4, Ad-SOCS3 or Ad-p53, respectively (n=3, average ± SE bars, **p< 0.01).

MDA-MB-231 cells were infected with the recombinant adenoviral vectors Ad-CD44-N-HIF3α4, Ad-SOCS3, and Ad-p53 *in vitro*, and real-time RT-PCR was conducted to assess whether their respective genes were efficiently transduced. The mRNA expressions of the *HIF-3a4* gene, the *SOCS3* gene, and the *p53* gene were significantly increased by infection with Ad-CD44-N-HIF3α4 (**Figure 6A**), Ad-SOCS3 (**Figure 6B**), and Ad-p53 (**Figure 6C**), respectively (p < 0.01).

### 3.6 Ad-CD44-N-HIF3α4 significantly suppressed CD44-downstream genes under culture conditions of hypoxia

**Figure 7:**
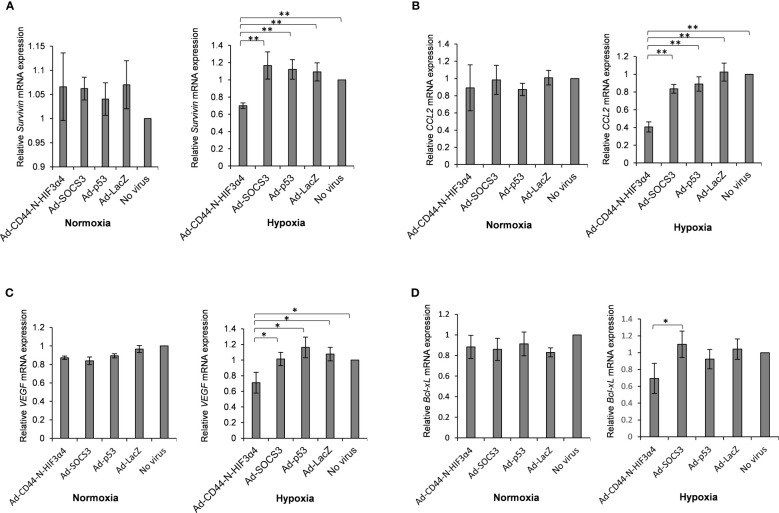
Gene expression of *survivin*, *CCL2*, *VEGF* and *Bcl-xL* in MDA-MB-231 cells infected with adenovirus vectors were measured by real-time RT-PCR. Cells were cultured under hypoxic conditions or under normoxic conditions. The significantly decreased mRNA expressions of **(A)** *survivin* and **(B)** *CCL2* were observed in cells infected with Ad-CD44-N-HIF3α4 compared to cells infected with the other adenovirus vectors and no virus only in the culture conditions under hypoxic. The significantly decreased mRNA expressions of **(C)** *VEGF* and **(D)** *Bcl-xL* were observed in cells infected with Ad-CD44-N-HIF3α4 compared to cells infected with the other adenovirus vectors and no virus only in the culture conditions under hypoxic. mRNA levels were standardized by the expression levels of control gene *TBP*. (n=3, average ± SE bars, *p< 0.05,**p< 0.01).

MDA-MB-231 cells were infected with the Ad-CD44-N-HIF3α4, Ad-SOCS3, Ad-p53 and Ad-LacZ adenoviral vectors *in vitro* to examine whether Ad-CD44-N-HIF3α4 could suppress the downstream genes of CD44 *via* the CD44 decoy receptor function of the synNotch receptor. The relative mRNA expressions of *survivin* and *CCL2*, downstream genes of CD44, in the cells infected with Ad-CD44-N-HIF3α4 were significantly lower than in cells infected with the other adenoviral vectors and controls cells under culture conditions of hypoxia (p < 0.01; **Figures 7A, B**). Conversely, no significant change was observed under culture conditions of normoxia (**Figures 7A, B**).

### 3.7 Ad-CD44-N-HIF3α4 significantly suppressed hypoxia target genes under hypoxia

The adenoviral vectors: MDA-MB-231 cells were infected with Ad-CD44-N-HIF3α4, Ad-SOCS3, Ad-p53 or Ad-LacZ *in vitro* to examine whether Ad-CD44-N-HIF3α4 could suppress hypoxia target genes *via* the function of HIF-3α4 released from the synNotch receptor of CD44-N-HIF3α4 fusion protein. Under culture conditions of hypoxia, Ad-CD44-N-HIF3α4 significantly decreased the mRNA expression of VEGF compared to the other adenoviral vectors or no infection (p < 0.05; **Figure 7C**), and the mRNA expression of *B-cell lymphoma-extra arge (Bcl-xL)* was significantly decreased in the cells infected with Ad-CD44-N-HIF-3α4 compared to cells infected with Ad-SOCS3 (p < 0.05; **Figure 7D**), while these changes were not observed under culture conditions of normoxia (**Figures 7C, D**). However, these changes are relatively small compared to the changes in *Survivin* and *CCL2* genes, which are the downstream signals of CD44.

### 3.8 Intratumoral injections of Ad-CD44-N-HIF3α4 induced CD44 overexpression in the cell membrane in MDA-MB-231 xenograft tumors *in vivo*

**Figure 8:**
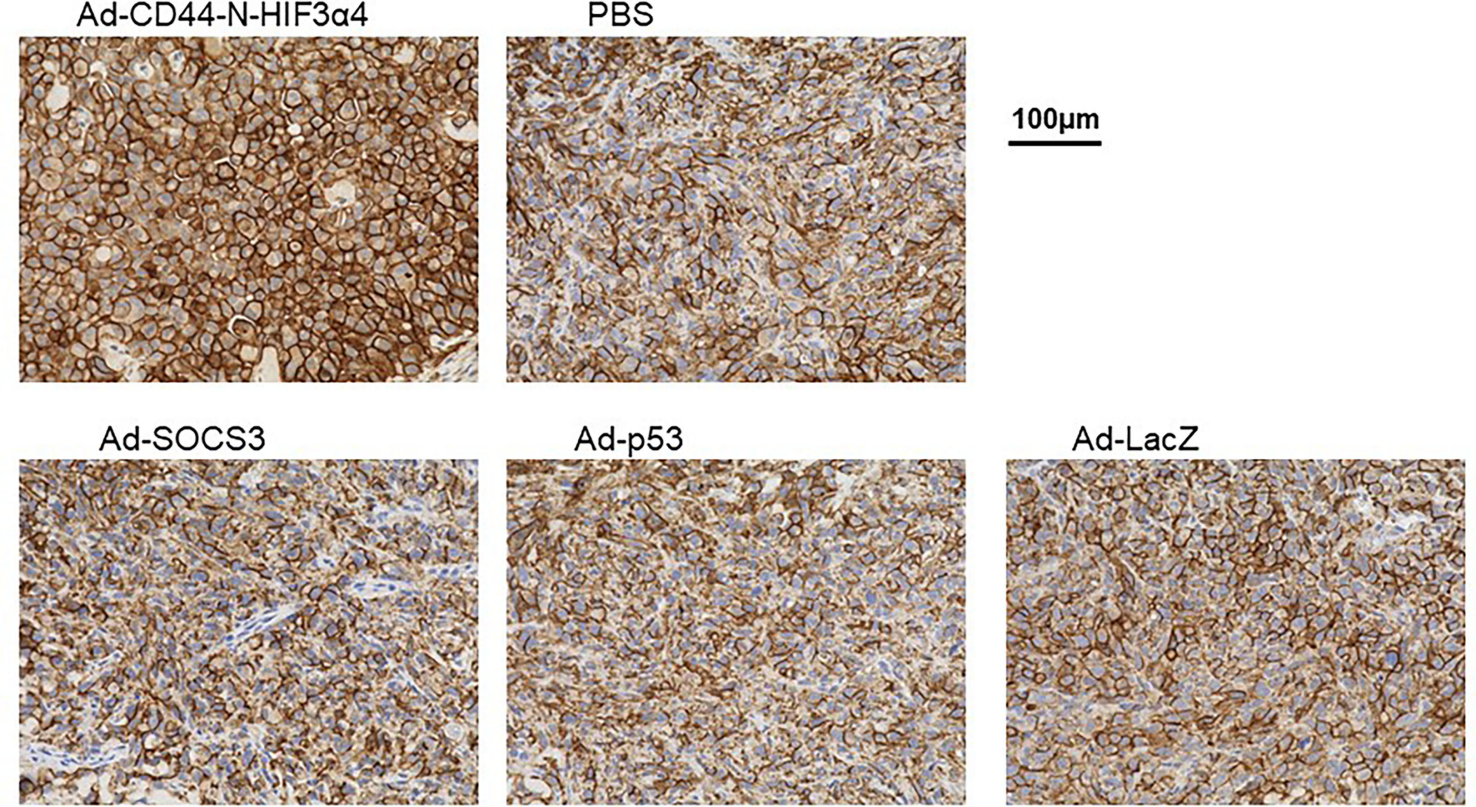
Immunohistochemical staining for CD44 in MDA-MB-231 tumor injected with adenovirus vectors One million MDA-MB-231 cells were subcutaneously inoculated into nude mice for tumor formation, followed by intratumoral injections of Ad-CD44-N-HIF3α4, Ad-SOCS3, Ad-p53, Ad-LacZ or PBS. After the treatments, tumors were resected and stained for CD44 expressions. The remarkably increased expression of CD44 was detected in the cell membrane of tumor injected with Ad-CD44-N-HIF3**α**4 compared to tumors injected with the other adenovirus vectors or PBS. (Original magnification: x400).

Immunohistochemical staining showed that Endogenous CD44 protein was positive in the cell membrane in MDA-MB-231 tumors in all treatment groups (**Figure 8**). However, the strongest signal was clearly observed in tumors treated with Ad-CD44-N-HIF3α4 (**Figure 8**). This result was consistent with the CD44 transduction by Ad-CD44-N-HIF3α4 confirmed with the western blotting (**Figure 4**), indicating that Ad-CD44-N-HIF3α4 could induce the overexpression of CD44-ECD at the cell surface of infected tumor cells.

### 3.9 Intratumoral injections of Ad-CD44-N-HIF3α4 significantly suppressed the growth of MDA-MB-231 xenograft tumors *in vivo*

**Figure 9:**
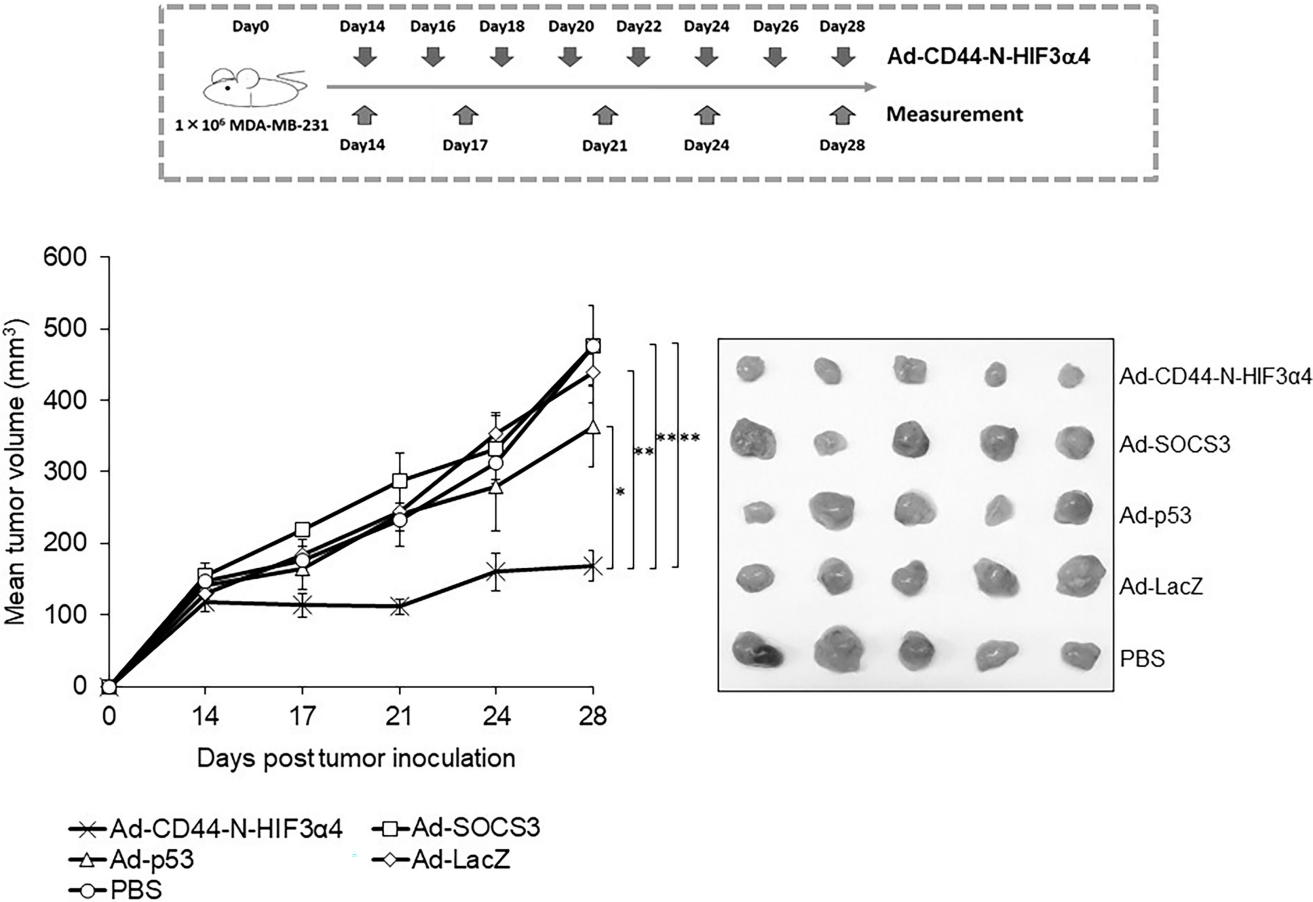
Anti-tumor effect of Ad-CD44-N-HIF3α4 in mice with MDA-MB-231 tumors One million MDA-MB-231 cells were subcutaneously inoculated into nude mice and intratumoral injections of Ad-CD44-N-HIF3**α**4, Ad-SOCS3, Ad-p53, Ad-LacZ or PBS were performed every other day for 8 times. Ad-CD44-N-HIF3α4 significantly inhibited the growth of MDA-MB-231 tumors compared to Ad-p53 (p< 0.05), Ad-SOCS3, Ad-LacZ and PBS (p< 0.01). (n=5, average ± SE bars, *p< 0.05, **p< 0.01).

The adenoviral vectors Ad-CD44-N-HIF3α4, Ad-SOCS3, Ad-p53 and Ad-LacZ or PBS were intratumorally injected into the MDA-MB-231 subcutaneous xenograft tumors to examine the *in vivo* anti-tumor activity of Ad-CD44-N-HIF3α4 in comparison with Ad-SOCS3 and Ad-p53. Briefly, the intratumoral injection of each adenoviral vector was initiated at Day 14 after the MDA-MB-231 cell inoculation. Subsequently, the adenoviral vectors or PBS were injected every other day for a total of 8 times. At Day 28, Ad-CD44-N-HIF3α4 significantly suppressed the tumor growth compared to the other adenoviral vectors or PBS ((p < 0.05 for Ad-p53, p<0.01 for the other groups; **Figure 9**). Ad-SOCS3 and Ad-p53 did not demonstrate significant *in vivo* anti-tumor activity compared to Ad-LacZ or PBS (**Figure 9**).

## 4 Discussion

In the present study, we constructed Ad-CD44-N-HIF3α4 containing the *synNotch receptor* gene, which encodes a fusion protein comprised of CD44-ECD acting as a CD44 decoy receptor and HIF-3α4, a dominant negative inhibitor of HIF-1α and HIF-2α, connected by the Notch core regulatory region (**Figures 1B, C**). Recently, synNotch receptors have been developed to generate synthetic Notch signaling pathways (43, 44). SynNotch receptors consist of the Notch regulatory core region with an appended extracellular recognition domain and synthetic intracellular transcriptional domain (45, 46). Previously, Roybal and colleagues (43) constructed engineered T cells with synNotch receptors, in which Notch-ECD was replaced with a single-chain variable fragment (scFv) against cancer antigens such as CD19 and HER2, and Notch-ICD was replaced with the Gal4 DNA binding domain fused to the tetrameric viral transcriptional activator domain, VP64 (47). The synNotch receptors of the engineered T cells could bind to the target antigens on the targeted cancer cells and sequentially activate transcription of the intended genes to induce the activation of T cells or cancer cell apoptosis (43).

To evaluate the anti-tumor activity of Ad-CD44-N-HIF3α4 in advanced breast cancer, we employed MDA-MB-231 cell line. Both MDA-MB-231 and MCF-7 (Luminal A, ER+, HER2-) cells expressed CAR and CD44, but the expression of CD44 proteins in MDA-MB-231 was higher than that in MCF-7 cells (**Figure 2A**). However, Cho and his colleagues reported that CD44-ICD is strongly expressed in both MAD-MB-231 and MCF-7 cells (48). Reportedly, the expression levels of CD44 protein in MDA-MB-231 and MCF7 cells are higher than that in MCF10A, which is normal brest cell line (49) Although hypoxia condition increased VEGF in both MDA-MB-231 and MCF-7 cells, HIF-1α was increase by hypoxia only in MDA-MB-231 but not in MCF-7 (**Figure 2B**). In addition, MDA-MB-231 but not MCF-7 cells express HAS2 (**Figure 3B**). This result is consistent with a previous report that the expression level of HAS2 in MDA-MB-231 is higher than MCF-7 and MCF10A cells (50). It is well known that HAS2 promotes breast cancer cell invasion through the CD44 pathway (41). Indeed, in our experiments, Ad-CD44-N-HIF3α4 could transduce CD44-ECD in both MDA-MB-231 and MCF-7 cells (**Figure 4A**), however, the cell growth was significantly inhibited by Ad-CD44-N-HIF3α4 only in MDA-MB-231 cells but not in MCF-7 (**Figure 4B**). These results suggested that Ad-CD44-N-HIF3α4 specifically worked in CD44 over expressing cancer cells. All taken together we selected MDA-MB-231 cells to evaluate the efficacy of Ad-CD44-N-HIF3α4 in advanced breast cancer. As well as inhibition of cell proliferation, we confirmed that Ad-CD44-N-HIF3α4 could inhibit the cell migration of MDA-MB-231 cells in hypoxic condition (**Figure 5**).

To evaluate the efficacy of Ad-CD44-N-HIF3α4, we compared it`s *in vivo* anti-tumor activities with other adenovirus vectors, Ad-SOCS3 (30) and Ad-p53 (31). In addition, the interaction between CD44 and HA enhances the infectivity of Ad-vectors (51). The suppressor of cytokine signaling (SOCS) family proteins are inhibitors of the Janus kinase/signal transducer and activator of transcription (JAK/STAT) signaling pathway. Higher expressions of SOCS1, 3, 4, and 7 are associated with good prognosis in breast cancer (52), and STAT3 is constitutively activated in breast cancer (53). Previously we demonstrated that Ad-SOCS3 could inhibit the growth of human and mouse prostate cancer cells *via* inhibition of interleukin-6 (IL-6)/JAK/STAT signaling (30). The *p53* gene mutation is frequently observed in TNBC cells, including the MDA-MB-231 cell line (54), and it is well known that Ad-p53 can increase the sensitivity to conventional cytotoxic agents (55, 56).

First, we confirmed that Ad-CD44-N-HIF3α4, Ad-SOCS3 and Ad-p53 could efficiently transduce *HIF-3α4*, *SOCS3* and *p53* genes in MDA-MB-231 cells (**Figures 6A–C**) respectively. To confirm the function of Ad-CD44-N-HIF3α4, we examined the function of Ad-CD44-N-HIF-3α4 under *in vitro* culture conditions of hypoxia in MDA-MB-231 cells. We confirmed that Ad-CD44-N-HIF3α4 significantly suppressed the mRNA expressions of *survivin* and *CCL2* genes, which are CD44-downstream target genes, compared to the other adenovirus vectors (**Figures 7A, B**). Survivin is an inhibitor of apoptosis protein that plays a pivotal role in stemness and invasion in breast cancer cells (57). Chemokine (C-C motif) ligand 2 (CCL2), a pro-inflammatory chemokine, has been implicated in breast cancer progression and the expression level of CCL2 is closely correlated with accumulation of tumor-associated macrophages (TAM) and breast cancer metastasis (58, 59). Also, we examined the effects of Ad-CD44-N-HIF3α4 on HIF target genes, *VEGF* and *Bcl-xL*. VEGF is an important growth factor for tumor angiogenesis, and it is considered as a specific target of HIF-1α (60). Generally, cytotoxic agents induce cancer cell apoptosis, but in some types of cancer cells, including breast cancer cells, anti-apoptotic proteins like Bcl-xL are also induced by cytotoxic agents (61). Chemoresistance induced by HIF could be implemented by anti-apoptotic proteins, including Bcl-xL (62). In our results, the expression of both *VEGF* and *Bcl-xL* genes was significantly suppressed by Ad-CD44-N-HIF3α4 compared to the other adenovirus vectors especially under culture condition of hypoxia (**Figures 7C, D**). Interestingly, these changes in both CD44 and HIF target genes were not observed under culture conditions of normoxia (**Figures 7A–D**). These results support the rationale for the function of Ad-CD44-N-HIF3α4.

In an *in vivo* study, we employed a mouse xenograft model of MDA-MB-231 tumor, of which hypoxia was previously confirmed by a three-dimensional multimodal molecular imaging with magnetic resonance (MR) imaging (63). The over-expression of CD44-ECD induced by Ad-CD44-N-HIF3α4 was clearly observed in immunohistochemical studies (**Figure 8**), and the anti-tumor activity of Ad-CD44-N-HIF3α4 was significantly greater than Ad-SOCS3 and Ad-p53 (**Figure 9**). Previously, both Ad-SOCS3 and Ad-p53 have demonstrated *in vivo* anti-tumor activity in many experimental models (35, 60, 64). However, in the xenograft model of MDA-MB-231, a highly invasive TNBC cell line, these adenovirus vectors could not suppress tumor growth. On the other hand, Ad-CD44-N-HIF3α4 did exert high anti-tumor activity in MDA-MB-231 tumors by targeting CD44 and HIF, both closely related to cancer stem cells. Also, we need to state the limitation of the xenograft model using an immune-deficient nude mice. While the multiple injections of Ad-vectors were performed here, the immune-responses to the vectors and transgenes could not be evaluated in this model. It is important to investigate in human clinical trials whether these immune-responses to Ad-vectors could enhance or reduce the anti-tumor activity. Also, in clinical setting of the treatment of TNBC, the sensitization to conventional cytotoxic agents by Ad-Vectors, especially Ad-p53 should be investigated (55, 56).

In conclusion, we developed a recombinant adenovirus vector, Ad-CD44-N-HIF3α4, containing a synNotch receptor gene which inhibited CD44 signaling and hypoxia-induced response in cancer cells. Ad-CD44-N-HIF3α4 worked only under conditions of hypoxia and the presence of HA *in vitro*, but greatly inhibited the growth of tumors of MDA-MB-231 invasive TNBC cells *in vivo*. These results indicate that Ad-CD44-N-HIF3α4 is a completely novel in vivo gene therapy drug targeting cancer stem cells, with potentially high clinical applicability for invasive types of cancer, especially TNBC.

## Data availability statement

The datasets presented in this article are not readily available because The dataset may be restricted because of confidentially promises or proprietaries. Requests to access the datasets should be directed to toshiro@med.kobe-u.ac.jp.

## Ethics statement

The animal study was reviewed and approved by the committees of the Kobe University Graduate School of Medicine.

## Author contributions

NK and TS designed the study. RA, ST, EH, SN, MU and RY performed the data analysis. RA, NK and TS wrote the original manuscript. HU and KK revised the manuscript. TS provided funding acquisition. MF and TS supervised the study. All authors contributed to the article and approved the submitted version.

## References

[1] Mehanna J Haddad FG Eid R Lambertini M Kourie HR. Triple-negative breast cancer: current perspective on the evolving therapeutic landscape. Int J Women Health (2019) 11:431–7. doi: 10.2147/IJWH.S178349 PMC6682754 31447592

[2] Alabdulkareem H Pinchinat T Khan S Landers A Christos P Simmons R. The impact of molecular subtype on breast cancer recurrence in young women treated with contemporary adjuvant therapy. Breast J (2018) 24:148–53. doi: 10.1111/tbj.12853 28707744

[3] Fragomeni SM Sciallis A Jeruss JS. Molecular subtypes and local-regional control of breast cancer. Surg Oncol Clin N Am (2018) 27:95–120. doi: 10.1016/j.soc.2017.08.005 29132568 PMC5715810

[4] Foulkes WD Smith IE Reis-Filho JS. Triple-negative breast cancer. N Engl J Med (2010) 363:1938–48. doi: 10.1056/NEJMra1001389 21067385

[5] Bianchini G Balko JM Mayer IA Sanders ME Gianni L. Triple-negative breast cancer: Challenges and opportunities of a heterogeneous disease. Nat Rev Clin Oncol (2016) 13:674–90. doi: 10.1038/nrclinonc.2016.66 PMC5461122 27184417

[6] Lebert JM Lester R Powell E Seal M McCarthy J. Advances in the systemic treatment of triple-negative breast cancer. Curr Oncol (2018) 25:S142–50. doi: 10.3747/co.25.3954 PMC6001760 29910657

[7] Liedtke C Mazouni C Hess KR André F Tordai A Mejia JA. Response to neoadjuvant therapy and long-term survival in patients with triple-negative breast cancer. J Clin Oncol (2008) 26:1275–81. doi: 10.1200/JCO.2007.14.4147 18250347

[8] Creighton CJ Li X Landis M Dixon JM Neumeister VM Sjolund A. Residual breast cancers after conventional therapy display mesenchymal as well as tumor-initiating features. Proc Natl Acad Sci U.S.A. (2009) 106:13820–5. doi: 10.1073/pnas.0905718106 PMC2720409 19666588

[9] Lee HE Kim JH Kim YJ Choi SY Kim SW Kang E. An increase in cancer stem cell population after primary systemic therapy is a poor prognostic factor in breast cancer. Br J Cancer (2011) 104:1730–8. doi: 10.1038/bjc.2011.159 PMC3111169 21559013

[10] Shima H Yamada A Ishikawa T Endo I. Are breast cancer stem cells the key to resolving clinical issues in breast cancer therapy? Gland Surg (2017) 6:82–8. doi: 10.21037/gs.2016.08.03 PMC5293654 28210556

[11] Al-Hajj M Wicha MS Benito-Hernandez A Morrison SJ Clarke MF. Prospective identification of tumorigenic breast cancer cells. Proc Natl Acad Sci U.S.A. (2003) 100:3983–8. doi: 10.1073/pnas.0530291100 PMC153034 12629218

[12] Sultan M Vidovic D Paine AS Huynh TT Coyle KM Thomas ML. Epigenetic silencing of TAP1 in aldefluor+ breast cancer stem cells contributes to their enhanced immune evasion. Stem Cells (2018) 36:641–54. doi: 10.1002/stem.2780 29341428

[13] Li W Ma H Zhang J Zhu L Wang C Yang Y. Unraveling the roles of CD44/CD24 and ALDH1 as cancer stem cell markers in tumorigenesis and metastasis. Sci Rep (2017) 7:13856. doi: 10.1038/s41598-017-14364-2 29062075 PMC5653849

[14] Bourguignon LY Zhu H Shao L Chen YW. CD44 interaction with c-src kinase promotes cortactin-mediated cytoskeleton function and hyaluronic acid-dependent ovarian tumor cell migration. J Biol Chem (2001) 276:7327–36. doi: 10.1074/jbc.M006498200 11084024

[15] Fujita Y Kitagawa M Nakamura S Azuma K Ishii G Higashi M. CD44 signaling through focal adhesion kinase and its anti-apoptotic effect. FEBS Lett (2002) 528:101–8. doi: 10.1016/S0014-5793(02)03262-3 12297287

[16] Bazil V Horejsí V. Shedding of the CD44 adhesion molecule from leukocytes induced by anti-CD44 monoclonal antibody simulating the effect of a natural receptor ligand. J Immunol (1992) 149:747–3. doi: 10.4049/jimmunol.149.3.747 1634766

[17] Murakami D Okamoto I Nagano O Kawano Y Tomita T Iwatsubo T. Presenilin-dependent gamma-secretase activity mediates the intramembranous cleavage of CD44. Oncogen (2003) 22:1511–6. doi: 10.1038/sj.onc.1206298 12629514

[18] Lu H Samanta D Xiang L Zhang H Hu H Chen I. Chemotherapy triggers HIF-1-dependent glutathione synthesis and copper chelation that induces the breast cancer stem cell phenotype. Proc Natl Acad Sci U.S.A. (2015) 112:E4600–9. doi: 10.1073/pnas.1513433112 PMC4547233 26229077

[19] Oliveira-Costa JP Zanetti JS Silveira GG Soave DF Oliveira LR Zorgetto VA. Differential expression of HIF-1α in CD44+CD24-/low breast ductal carcinomas. Diagn Pathol (2011) 6:73. doi: 10.1186/1746-1596-6-73 21824412 PMC3170242

[20] Li Z Bao S Wu Q Wang H Eyler C Sathornsumetee S. Hypoxia-inducible factors regulate tumorigenic capacity of glioma stem cells. Cancer Cell (2009) 15:501–13. doi: 10.1016/j.ccr.2009.03.018 PMC2693960 19477429

[21] Semenza GL. The hypoxic tumor microenvironment: A driving force for breast cancer progression. Biochim Biophys Acta (2016) 1863:382–91. doi: 10.1016/j.bbamcr.2015.05.036 PMC4678039 26079100

[22] Vadde R Vemula S Jinka R Merchant N Bramhachari PV Nagaraju GP. Role of hypoxia-inducible factors (HIF) in the maintenance of stemness and malignancy of colorectal cancer. Crit Rev Oncol Hematol (2017) 113:22–7. doi: 10.1016/j.critrevonc.2017.02.025 28427511

[23] Kaelin WG Jr Ratcliffe PJ. Oxygen sensing by metazoans: the central role of the HIF hydroxylase pathway. Mol Cell (2008) 30:393–402. doi: 10.1016/j.molcel.2008.04.009 18498744

[24] Nguyen LV Vanner R Dirks P Eaves CJ. Cancer stem cells: an evolving concept. Nat Rev Cancer (2012) 12:133–43. doi: 10.1038/nrc3184 22237392

[25] Liu M Liu Y Deng L Wang D He X Zhou L. Transcriptional profiles of different states of cancer stem cells in triple-negative breast cancer. Mol Cancer (2018) 17:65. doi: 10.1186/s12943-018-0809-x 29471829 PMC5824475

[26] Kijima T Osaki T Nishino K Kumagai T Funakoshi T Goto H. Application of the cre recombinase/loxP system further enhances antitumor effects in cell type-specific gene therapy against carcinoembryonic antigen-producing cancer. Cancer Res (1999) 59:4906–11. 10519403

[27] Semenza GL. Dynamic regulation of stem cell specification and maintenance by hypoxia-inducible factors. Mol Asp Med (2016) 47-48:15–23. doi: 10.1016/j.mam.2015.09.004 26549347

[28] Bai J Chen WB Zhang XY Kang XN Jin LJ Zhang H. HIF-2α regulates CD44 to promote cancer stem cell activation in triple-negative breast cancer *via* PI3K/AKT/mTOR signaling. World J Stem Cells (2020) 12:87–99. doi: 10.4252/wjsc.v12.i1.87 32110277 PMC7031759

[29] Kopan R Ilagan MX. The canonical notch signaling pathway: unfolding the activation mechanism. Cell (2009) 137:216–33. doi: 10.1016/j.cell.2009.03.045 PMC2827930 19379690

[30] Yoneda T Kunimura N Kitagawa K Fukui Y Saito H Narikiyo K. Overexpression of SOCS3 mediated by adenovirus vector in mouse and human castration-resistant prostate cancer cells increases the sensitivity to NK cells *in vitro* and in vivo. Cancer Gene Ther (2019) 26:388–99. doi: 10.1038/s41417-018-0075-5 30607005

[31] Wang X Song H Yu Q Liu Q Wang L Liu Z. Ad-p53 enhances the sensitivity of triple-negative breast cancer MDA-MB-468 cells to the EGFR inhibitor gefitinib. Oncol Rep (2015) 33:526–32. doi: 10.3892/or.2014.3665 PMC4306269 25501339

[32] Shona T Graeme J. A cancer gene therapy approach that targets tumor-associated hyaluronan. Cancer Growth Metastasis (2009) 2:29–43. doi: 10.4137/CGM.S3716

[33] Morsut L Roybal KT Xiong X Gordley RM Coyle SM Thomson M. Engineering customized cell sensing and response behaviors using synthetic notch receptors. Cell (2016) 164:780–91. doi: 10.1016/j.cell.2016.01.012 PMC4752866 26830878

[34] Maynard MA Evans AJ Hosomi T Hara S Jewett MA Ohh M. Human HIF-3alpha4 is a dominant-negative regulator of HIF-1 and is down-regulated in renal cell carcinoma. FASEB J (2005) 19:191396–406. doi: 10.1096/fj.05-3788com 16126907

[35] Iwahori K Serada S Fujimoto M Nomura S Osaki T Lee CM. Overexpression of SOCS3 exhibits preclinical antitumor activity against malignant pleural mesothelioma. Int J Cancer (2011) 129:1005–17. doi: 10.1002/ijc.25716 20949562

[36] Miyake S Makimura M Kanegae Y Harada S Sato Y Takamori K. Efficient generation of recombinant adenoviruses using adenovirus DNAterminal protein complex and a cosmid bearing the full-length virus genome. Proc Natl Acad Sci USA (1996) 93:1320–24. doi: 10.1073/pnas.93.3.1320 PMC40078 8577762

[37] Saito H Kitagawa K Yoneda T Fukui Y Fujsawa M Bautista D. Combination of p53-DC vaccine and rAd-p53 gene therapy induced CTLs cytotoxic against p53-deleted human prostate cancer cells *in vitro* . Cancer Gene Ther (2017) 24:289–96. doi: 10.1038/cgt.2017.21 28621316

[38] Goto H Osaki T Kijima T Nishino K Kumagai T Funakoshi T. Gene therapy utilizing the Cre/loxP system selectively suppresses tumor growth of disseminated carcinoembryonic antigen-producing cancer cells. Int J Cancer (2001) 94:414–19. doi: 10.1002/ijc.1474 11745423

[39] Bray S Musisi H Bienz M. Bre1 is required for notch signaling and histone modification. Dev Cell (2005) 8:279–86. doi: 10.1016/j.devcel.2004.11.020 15691768

[40] Bergelson JM Cunningham JA Droguett G Kurt-Jones EA Krithivas A Hong JS. Isolation of a common receptor for coxsackie b viruses and adenoviruses 2 and 5. Science (1997) 275:1320–3. doi: 10.1126/science.275.5304.1320 9036860

[41] Bernert B Porsch H Heldin P. Hyaluronan synthase 2 (HAS2) promotes breast cancer cell invasion by suppression of tissue metalloproteinase inhibitor 1 (TIMP-1). J Biol Chem (2011) 286:42349–59. doi: 10.1074/jbc.M111.278598 PMC3234988 22016393

[42] Bytautaite M Petrikaite V. Comparative study of lipophilic statin activity in 2D and 3D *in vitro* models of human breast cancer cell lines MDA-MB-231 and MCF-7. Onco Targets Ther (2020) 13:13201–9. doi: 10.2147/OTT.S283033 PMC7769197 33380809

[43] Roybal KT Williams JZ Morsut L Rupp LJ Kolinko I Choe JH. Engineering T cells with customized therapeutic response programs using synthetic notch receptors. Cell (2016) 167:419–32.e16. doi: 10.1016/j.cell.2016.09.011 27693353 PMC5072533

[44] Roybal KT Rupp LJ Morsut L Walker WJ McNally KA Park JS. Precision tumor recognition by T cells with combinatorial antigen-sensing circuits. Cell (2016) 164:770–9. doi: 10.1016/j.cell.2016.01.011 PMC4752902 26830879

[45] Gordon WR Zimmerman B He L Miles LJ Huang J Tiyanont K. Mechanical allostery: Evidence for a force requirement in the proteolytic activation of notch. Dev Cell (2015) 33:729–36. doi: 10.1016/j.devcel.2015.05.004 PMC4481192 26051539

[46] Mueller SN Gebhardt T Carbone FR Heath WR. Memory T cell subsets, migration patterns, and tissue residence. Annu Rev Immunol (2013) 31:137–61. doi: 10.1146/annurev-immunol-032712-095954 23215646

[47] Lecourtois M Schweisguth F. Indirect evidence for delta-dependent intracellular processing of notch in drosophila embryos. Curr Biol (1998) 8:771–4. doi: 10.1016/S0960-9822(98)70300-8 9651681

[48] Cho Y Lee H-W Kang HG Kim HY Kim SJ Chun KH. Cleaved CD44 intracellular domain supports activation of stemness factors and promotes tumorigenesis of breast cancer. Oncotarget (2015) 6:8709–21. doi: 10.18632/oncotarget.3325 PMC4496178 25909162

[49] Hamilton SR Fard SF Painwand FF Tolg C Veiseh M Wang C. The hyaluronan receptors CD44 and RHAMM(CD168) from complexes with ERK1,2, which sustains high basal motility in breast cancer cells. J Biol Chem (2007) 282:16667–80. doi: 10.1074/jbc.M702078200 PMC2949353 17392272

[50] Wu M Cao M He Y Liu Y Yang C Du Y. A novel role of low molecular weight hyaluronan in breast cancer metastasis. FASEB J (2015) 29:1290–8. doi: 10.1096/fj.14-259978 25550464

[51] Chaudhuri SR Mallam JN Chévez-Barrios P Wadhwa L Ng P Hurwitz MY. Modulation of adenoviral transduction *in vitro* and *in vivo* by hyaluronan and its receptor CD44. Mol Ther (2007) 15:566–70. doi: 10.1038/sj.mt.6300044 17180120

[52] Sasi W Jiang WG Sharma A Mokbel K. Higher expression levels of SOCS 1,3,4,7 are associated with earlier tumour stage and better clinical outcome in human breast cancer. BMC Cancer (2010) 10:178. doi: 10.1186/1471-2407-10-178 20433750 PMC2876081

[53] Banerjee K Resat H. Constitutive activation of STAT3 in breast cancer cells: A review. Int J Cancer (2016) 138:2570–8. doi: 10.1002/ijc.29923 PMC4801660 26559373

[54] Jones KR Elmore LW Jackson-Cook C Demasters G Povirk LF Holt SE. p53-dependent accelerated senescence induced by ionizing radiation in breast tumour cells. Int J Radiat Biol (2005) 81:445–58. doi: 10.1080/09553000500168549 16308915

[55] Shirakawa T Sasaki R Gardner TA Kao C Zhang ZJ Sugimura K. Drug-resistant human bladder-cancer cells are more sensitive to adenovirus-mediated wild-type p53 gene therapy compared to drug-sensitive cells. Int J Cancer (2001) 94:282–9. doi: 10.1002/ijc.1453 11668510

[56] Inoue A Narumi K Matsubara N Sugawara S Saijo Y Satoh K. Administration of wild-type p53 adenoviral vector synergistically enhances the cytotoxicity of anti-cancer drugs in human lung cancer cells irrespective of the status of p53 gene. Cancer Lett (2000) 157:105–12. doi: 10.1016/S0304-3835(00)00480-8 10893449

[57] Lin W Xie J Xu N Huang L Xu A Li H. Glaucocalyxin a induces G2/M cell cycle arrest and apoptosis through the PI3K/Akt pathway in human bladder cancer cells. Int J Biol Sci (2018) 14:418–26. doi: 10.7150/ijbs.23602 PMC5930474 29725263

[58] Ueno T Toi M Saji H Muta M Bando H Kuroi K. Significance of macrophage chemoattractant protein-1 in macrophage recruitment, angiogenesis, and survival in human breast cancer. Clin Cancer Res (2000) 6:3282–9. 10955814

[59] Saji H Koike M Yamori T Saji S Seiki M Matsushima K. Significant correlation of monocyte chemoattractant protein-1 expression with neovascularization and progression of breast carcinoma. Cancer (2001) 92:1085–91. doi: 10.1002/1097-0142(20010901)92:5<1085::AID-CNCR1424>3.0.CO;2-K 11571719

[60] Kachamakova-Trojanowska N Podkalicka P Bogacz T Barwacz S Józkowicz A Dulak J. HIF-1 stabilization exerts anticancer effects in breast cancer cells *in vitro* and in vivo. Biochem Pharmacol (2020) 175:113922. doi: 10.1016/j.bcp.2020.113922 32205093

[61] Fiebig AA Zhu W Hollerbach C Leber B Andrews DW. Bcl-XL is qualitatively different from and ten times more effective than bcl-2 when expressed in a breast cancer cell line. BMC Cancer (2006) 6:213. doi: 10.1186/1471-2407-6-213 16928273 PMC1560389

[62] Okazaki M Fushida S Tsukada T Kinoshita J Oyama K Miyashita T. The effect of HIF-1α and PKM1 expression on acquisition of chemoresistance. Cancer Manag Res (2018) 10:1865–74. doi: 10.2147/CMAR.S166136 PMC6037278 30013393

[63] Jiang L Greenwood TR Artemov D Raman V Winnard PT Jr Heeren RM. Localized hypoxia results in spatially heterogeneous metabolic signatures in breast tumor models. Neoplasia (2012) 14:732–41. doi: 10.1593/neo.12858 PMC3431180 22952426

[64] Li Y He W Wang R Yang L Zhou C Zhang B. Antitumor effects of recombinant human adenovirus-p53 against human cutaneous squamous cell carcinoma in mice. Exp Ther Med (2016) 12:4159–67. doi: 10.3892/etm.2016.3901 PMC5228342 28105142

